# Smart bone plates can monitor fracture healing

**DOI:** 10.1038/s41598-018-37784-0

**Published:** 2019-02-14

**Authors:** Monica C. Lin, Diane Hu, Meir Marmor, Safa T. Herfat, Chelsea S. Bahney, Michel M. Maharbiz

**Affiliations:** 1 Department of Bioengineering, University of California, Berkeley, CA 94720 USA; 2 UCSF Orthopaedic Trauma Institute, Zuckerberg San Francisco General Hospital, San Francisco, CA 94110 USA; 3 Center for Regenerative Sports Medicine, Steadman Philippon Research Institute, Vail, CO 81657 USA; 4 Department of Electrical Engineering and Computer Science, University of California, Berkeley, CA 94720 USA; 5 Chan Zuckerberg Biohub, San Francisco, CA 94158 USA

## Abstract

There are currently no standardized methods for assessing fracture healing, with physicians relying on X-rays which are only useful at later stages of repair. Using *in vivo* mouse fracture models, we present the first evidence that microscale instrumented implants provide a route for post-operative fracture monitoring, utilizing electrical impedance spectroscopy (EIS) to track the healing tissue with high sensitivity. In this study, we fixed mouse long bone fractures with external fixators and bone plates. EIS measurements taken across two microelectrodes within the fracture gap were able to track longitudinal differences between individual mice with good versus poor healing. We additionally present an equivalent circuit model that combines the EIS data to classify fracture repair states. Lastly, we show that EIS measurements strongly correlated with standard quantitative µCT values and that these correlations validate clinically-relevant operating frequencies for implementation of this technique. These results demonstrate that EIS can be integrated into current fracture management strategies such as bone plating, providing physicians with quantitative information about the state of fracture repair to guide clinical decision-making for patients.

## Introduction

Musculoskeletal injuries are among the most disabling conditions in the United States, with the total number of bone fractures ranging from 12 to 15 million per year^1^. Treatment of these fractures represents a significant burden on the U.S. healthcare system, with hospitalization costing an estimated $23.4 billion in 2004^2^. Determining how well a fracture is healing is crucial to making correct clinical decisions for patients, but multiple studies cite the lack of standardized methods for assessing fracture union^3–6^.

Radiographic imaging and physical evaluation are two common methods of monitoring bone fracture healing in the clinic. Plain X-ray radiographs are often used, but studies have shown that these correlate poorly with bone strength, do not define union with enough accuracy, and are unreliable for determining the stage of fracture repair^7^. Computed tomography (CT), dual energy X-ray absorptiometry (DEXA), and ultrasound can offer improved diagnostic capabilities, but have limited use in the clinic primarily due to cost and high radiation dose^7,8^. Physical examinations by a physician are often relied upon, but these are subjective and can result in imprecise assessments^3^.

Fracture healing proceeds through a combination of two pathways: intramembranous (direct) and endochondral (indirect) ossification^9,10^. At the onset of fracture injury, formation of a hematoma initiates a pro-inflammatory cascade (Stage 1)^11,12^. Following this, endosteal and periosteal progenitor cells undergo intramembranous ossification to directly form bone in the highly stable areas^13,14^. Within the fracture gap, where there is more motion, new bone forms indirectly from a cartilage intermediate through the process of endochondral ossification (Stage 2)^13,15^. Hypertrophic maturation of chondrocytes then promotes mineralization and leads to conversion of cartilage into trabecular bone (Stage 3)^16^, where it is then remodeled into functional cortical bone (Stage 4). These four defined stages of healing are well characterized histologically^10,16,17^, but early stages in particular are not detectable by typical methods of monitoring like X-ray that rely on mineralization of bone.

Monitoring fracture healing is an area of active academic research, but most work has focused around obtaining mechanical feedback correlating strain measurements to bone strength^18,19^. In this study, we utilize electrical techniques to characterize progression of fracture repair, building on previous work that has measured electrical changes in cells and tissue^20–22^. Electrically, tissue can be modelled as a combination of resistive and capacitive effects. The ion-rich intra- and extracellular matrices conduct charge and thus can be modeled as resistances, while double-layered cell membranes act as barriers to charge flow and can be modeled as capacitances or constant phase elements (CPE)^23^. Electrical impedance spectroscopy (EIS) measures the frequency-dependent combination of these components, describing opposition to the flow of electrical current through a material. Complex impedance *Z* can be expressed as

1 $$Z=R+{\rm{j}}X$$

with resistance R and reactance X.

There is an extensive body of literature^23–26^ demonstrating the use of this so-called “bioimpedance” method to quantitatively characterize cellular changes, primarily reflecting cell membrane integrity, cell volumes, and the conductivity of intra- and extracellular components^26^. Previous work has detailed the use of EIS to describe many biological tissues^26–29^, including the dielectric and conductive properties of porous and dense bone^30–32^. We have also shown use of EIS to distinguish tissue composition within the fracture callus in *ex vivo* model systems^33,34^. While prior studies have utilized EIS to evaluate bone fractures in *ex vivo* and *in vivo* models^35–40^, they suffer from restricted detection due to noise from surrounding soft tissue, limited sensitivity due to electrode placement, and inadequate histological analysis.

Here, we present the development and testing of microscale EIS sensors designed to measure electrical properties of the fracture callus longitudinally during healing in two different murine fracture models. To our knowledge, this is the first study to implant microscale sensors directly in the fracture gap, enabling local measurements of the changing callus. By doing this, we demonstrated the ability of our sensors to distinguish between healing and poor-healing fractures stabilized using miniaturized external fixators or bone plates, and found that frequency spectra of impedance measurements are robustly correlated with quantified measures of bone volume and bone mineral density. The long-term vision is that these EIS sensors can be used to augment current clinical care by quantitatively monitoring the progression of healing through regular measurements at the fracture site, and further enable early assessment of risk for nonunion. With the global orthopaedic devices market expected to reach $41.2 billion by 2019^41^, we believe there is vast potential for a smart implant system that can integrate with existing orthopaedic hardware platforms to provide physicians with information about each patient’s individual healing trajectory. Our proof-of-concept study is an important step in supporting the feasibility of EIS as a simple and low-cost method to provide clinically-relevant information during fracture management.

## Results

### Impedance distinguishes fractures vs. critical-sized defects

**Figure 1:**
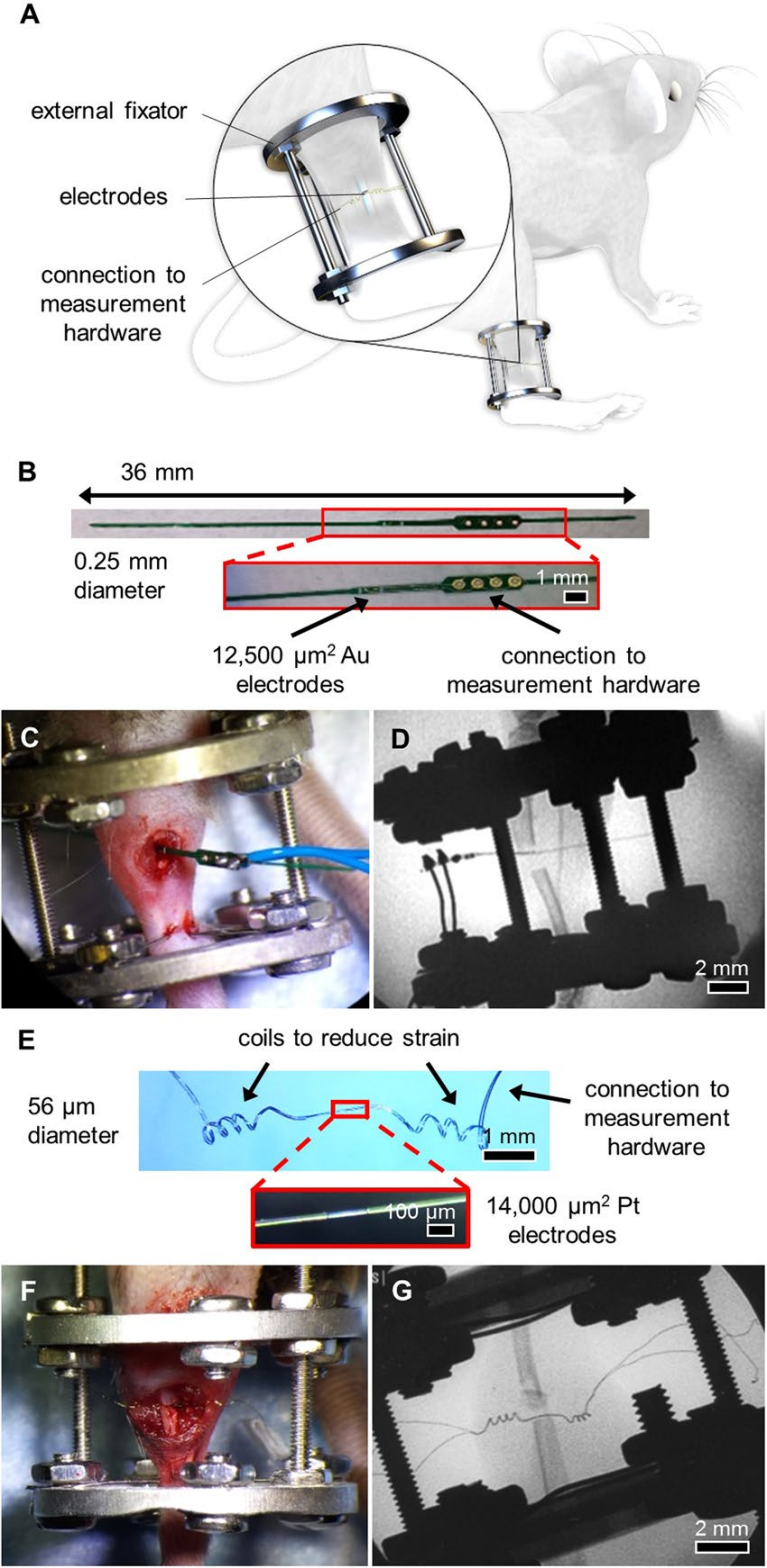
System overview and sensors for an external fixator model. (**A**) System overview of sensor embedded in a mouse tibia fracture, where the injury is stabilized with an external fixator. Image created by the Ella Maru Studio. (**B**) 0.25 mm diameter sensor fabricated on an FR4 substrate, with gold (Au) surface electrodes and large vias outside the leg to connect to measurement hardware. Sensors were implanted in externally-fixed mice tibias with 0.5 mm and 2 mm defects. (**C**) Photograph of open surgery performed to implant 0.25 mm sensor in the external fixator model. Surgical site was closed following sensor placement. (**D**) Fluoroscopy image of implanted 0.25 mm sensor in a 2 mm defect. (**E**) 56 µm diameter sensor assembled using platinum (Pt) wire, with recording sites exposed by a CO_2_ laser and coils added to provide strain relief. (**F**) Photograph of open surgery performed to implant 56 µm sensor in the external fixator model. Surgical site was closed following sensor placement. (**G**) Fluoroscopy image of implanted 56 µm sensor in a 0.5 mm defect.

**Figure 2:**
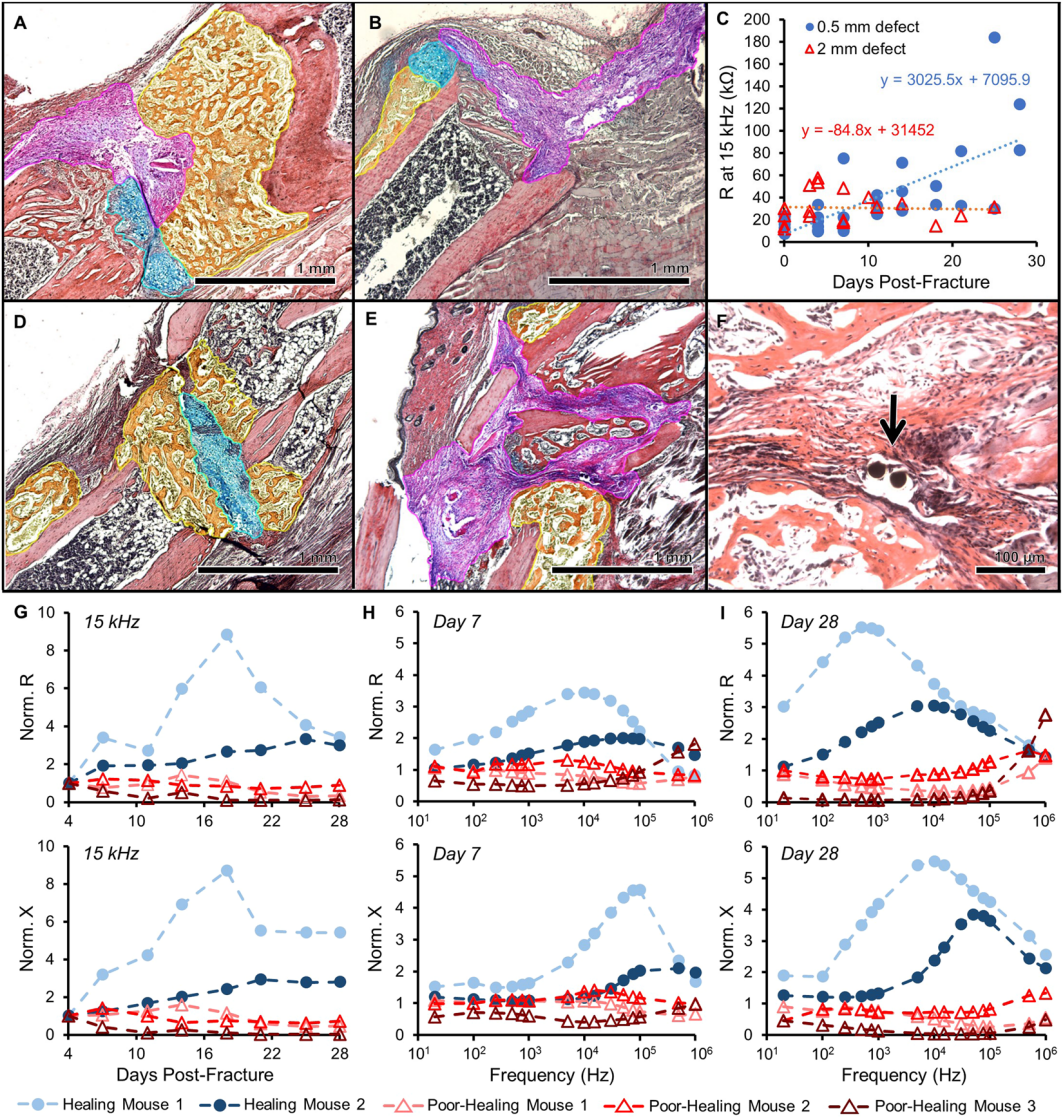
Impedance data distinguishes healing and poorly healing tibia fractures in an external fixator model. Histology sections in this image are stained with Hall’s and Brunt’s Quadruple (HBQ) stain and false-colored to aid interpretation of tissue composition. Blue = cartilage, yellow = trabecular bone, and purple = fibrous/amorphous tissue. Original red color = cortical bone, black/white area = bone marrow. (**A**) Representative histology section for an externally-fixed 0.5 mm defect at 14 days post-fracture; the fracture gap is clearly bridged by cartilage and new trabecular bone. (**B**) Representative histology section for an externally-fixed 2 mm critical-sized defect at 14 days post-fracture; the fracture gap is dominated by fibrous tissue. (**C**) Electrical resistance (R) at 15 kHz measured with a 250 µm sensor is plotted over days post-fracture for measurements taken in mice with 0.5 mm (N = 6) and 2 mm (N = 5) defects. Linear regression analyses determined that there is a significant positive relationship in mice with 0.5 mm defects (p < 0.0001), while there is no correlative relationship in mice with 2 mm defects. (**D**) Representative histology section for a healing mouse at 28 days post-fracture; the fracture gap is clearly bridged by cartilage and new trabecular bone. (**E**) Representative histology section for a poor-healing mouse at 28 days post-fracture; the fracture gap contains an overabundance of fibrous tissue. (**F**) Black arrow points to 56 µm sensor fully embedded in fracture tissue. (**G**) Electrical resistance (R) and reactance (X) normalized as a ratio to day 4 plotted over the course of fracture healing at 15 kHz. Normalized R and X both rise steadily over healing time in the healing mice, with stagnant values observed in the poor-healing mice. (**H**) Normalized R and X as a ratio to day 4 plotted over a range of frequencies at day 7 post-fracture. (**I**) Normalized R and X as a ratio to day 4 plotted over a range of frequencies at day 28 post-fracture. Marked shifts in frequency response from day 7 were observed in the healing mice, with limited change occurring in the poor-healing mice.

To measure impedance of a changing fracture callus over the course of healing, we implanted commercially-fabricated sensors into broken tibias stabilized in an external fixator model (Fig. 1A) for initial validation. These 250 µm diameter FR4 (a glass-reinforced epoxy laminate material) sensor pins (Fig. 1B–D) were placed into either 0.5 mm (N = 6) or 2 mm (N = 5) defects to determine if we could distinguish EIS trends between previously established healing versus non-healing fracture models, respectively^42–44^. Measurements were taken twice weekly from 20 Hz to 1 MHz with mice sacrificed at scattered time points up to day 28. Mice with 0.5 mm defects initiated a healing response with fracture calli containing a heterogeneous mixture of cartilage and new trabecular bone 14 days post-injury (Fig. 2A). However, mice with 2 mm defects contained primarily undifferentiated fibrous tissue indicating minimal healing response (Fig. 2B). Histology images have been false-colored to aid in interpretation of tissue composition (original histology images in Fig. S1A,B). Linear regression analyses indicate a significant positive relationship between electrical resistance (R) and time in mice with 0.5 mm defects (p < 0.0001), and no correlative relationship between R and time in mice with 2 mm defects (Fig. 2C). By day 28, histology for the 0.5 mm defects show new bone formation, while 2 mm critical-sized defects result in atrophic nonunions with absence of bony bridging.

### Microscale sensors track changes in frequency response

A limitation of the FR4 sensor was the repeated observation of fibrous tissue around the electrodes even out to 28 days post-injury. We hypothesized this was due to the sensor’s large size relative to the defect. Consequently, we designed and built significantly smaller sensors made from 56 µm diameter platinum (Pt) wire (Fig. 1E)^45^. Sensors were implanted within fractures stabilized in the same external fixator model (Fig. 1F,G) with 0.5 mm defects and we again measured EIS from 20 Hz to 1 MHz twice weekly for 28 days in five mice.

In two mice, histology showed clear signs of healing, with a robust callus present between the two bone ends (Figs 2D and S1C). However, the other three mice displayed poor signs of healing, with fracture gaps dominated by an overabundance of fibrous tissue (Figs 2E and S1D). We believe the divergent healing responses were due to significant sensor movement in some fractures based on fluoroscopy images taken immediately after surgery and on day 28 before euthanasia.

The thin and flexible nature of the sensors also allowed them to be preserved throughout the histology process, enabling visualization of the specific tissue surrounding the sensor (Fig. 2F). To analyze EIS data alongside histological evidence of healing, electrical resistance (R) and reactance (X) were normalized as a ratio to the first time point after surgery (day 4). This accounted for the initial perturbation of surgery and variation between sensors and samples, enabling comparison between multiple mice. Based on all EIS plots, we found the largest spread between measurement days at 15 kHz, indicating the most functional frequency for detecting differences in fracture healing (Fig. 2G). This falls within the frequency range that can be identified as the beta dispersion, which is associated with the polarization of cell membranes, proteins, and other macromolecules^26^. At this frequency, normalized R and X increase with progression of fracture repair in the healing mice, but stall in the poor-healing mice. Figure 2H,I illustrate how normalized R and X as functions of frequency differ for healing and poor-healing mice from an early time point (day 7) to a late time point (day 28). The healing mice exhibit clear changes in their frequency response in both parameters relative to day 4, especially from 10^3^ to 10^5^ Hz. Comparatively, poor-healing mice show relatively flat frequency responses relative to day 4. The influence of callus formation on frequency response enables the clear differentiation between well-healing and poor-healing mice.

### Impedance discriminates range of fracture states

**Figure 3:**
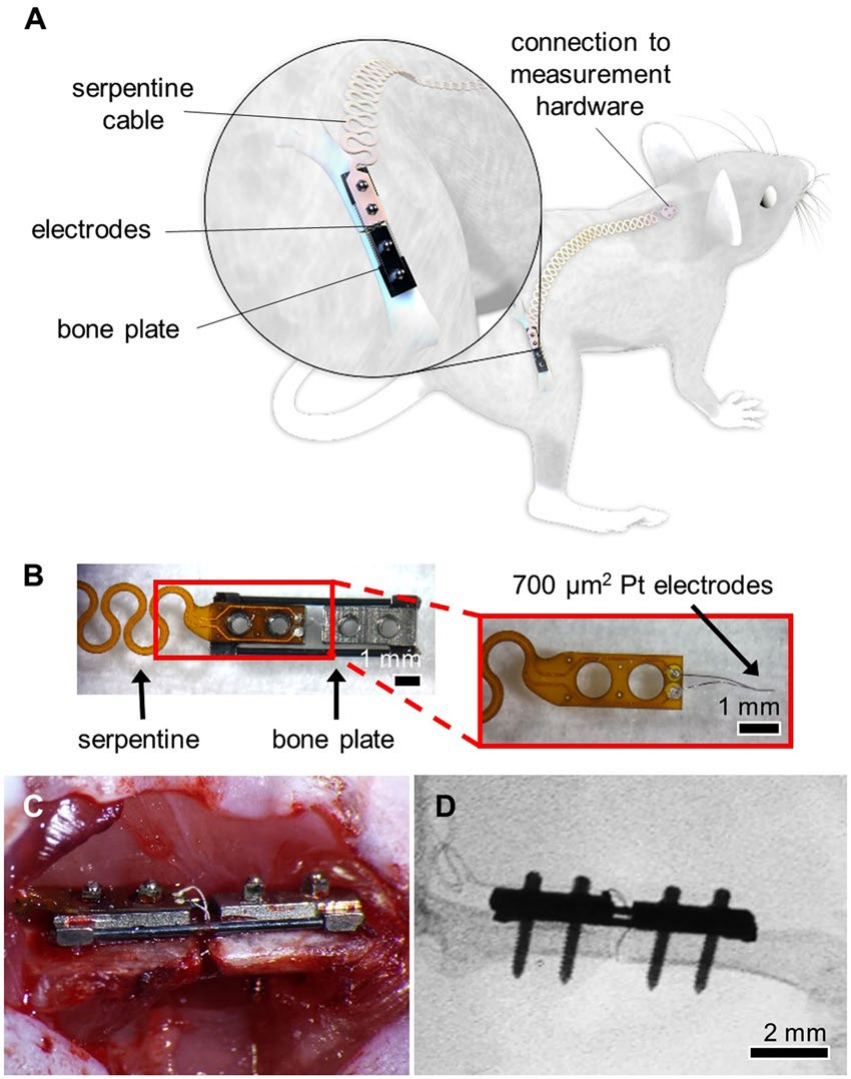
System overview and sensor for a bone plate model. (**A**) System overview of sensor embedded in a mouse femur fracture, where the injury is stabilized with a bone plate. Image created by the Ella Maru Studio. (**B**) Sensors fabricated on a polyimide substrate with 700 µm^2^ platinum (Pt) electrodes spaced 0.5 mm apart. Sensors were affixed to the proximal half of the bone plate, with a long flexible cable extending off the proximal end. The serpentine pattern repeated for the length of the cable, ending in two vias that served as connectors that link to the measurement hardware. (**C**) Photograph of open surgery performed to implant a bone plate and affixed sensor to stabilize a femur fracture. Surgical site was closed following sensor placement. (**D**) Fluoroscopy image of implanted Pt sensor in the fracture gap.

To extend utility of our monitoring technique to another clinically-relevant fixation procedure, we designed and built sensors for integration with a bone plate model. Bone plates are commonly used for patients with traumatic fractures requiring surgical intervention, and provided a platform for much shorter sensors that experience significantly less movement. We used flexible polyimide boards affixed to commercially available murine bone plates, attaching 25 µm diameter platinum wires as electrodes within the fracture gap (Fig. 3A,B). A long, serpentine cable extended from the proximal end of the plate and was tunneled subcutaneously to connection pads that interfaced with our measurement instrumentation. An open surgery was performed to affix these “smart” bone plates to the mouse femur, and electrodes were inserted into a <0.25 mm fracture gap (Fig. 3C,D). Surgery was also performed on unfractured control mice, placing the sensor between the bone and muscle. Impedance measurements were taken from 20 Hz to 1 MHz between two 700 µm^2^ electrodes three times weekly (Fig. S2), with mice euthanized at 12 (N = 3 fracture) or 26 (N = 5 fracture, N = 2 control) days post-surgery for histology and micro-Computed Tomography (µCT) analysis.

**Figure 4:**
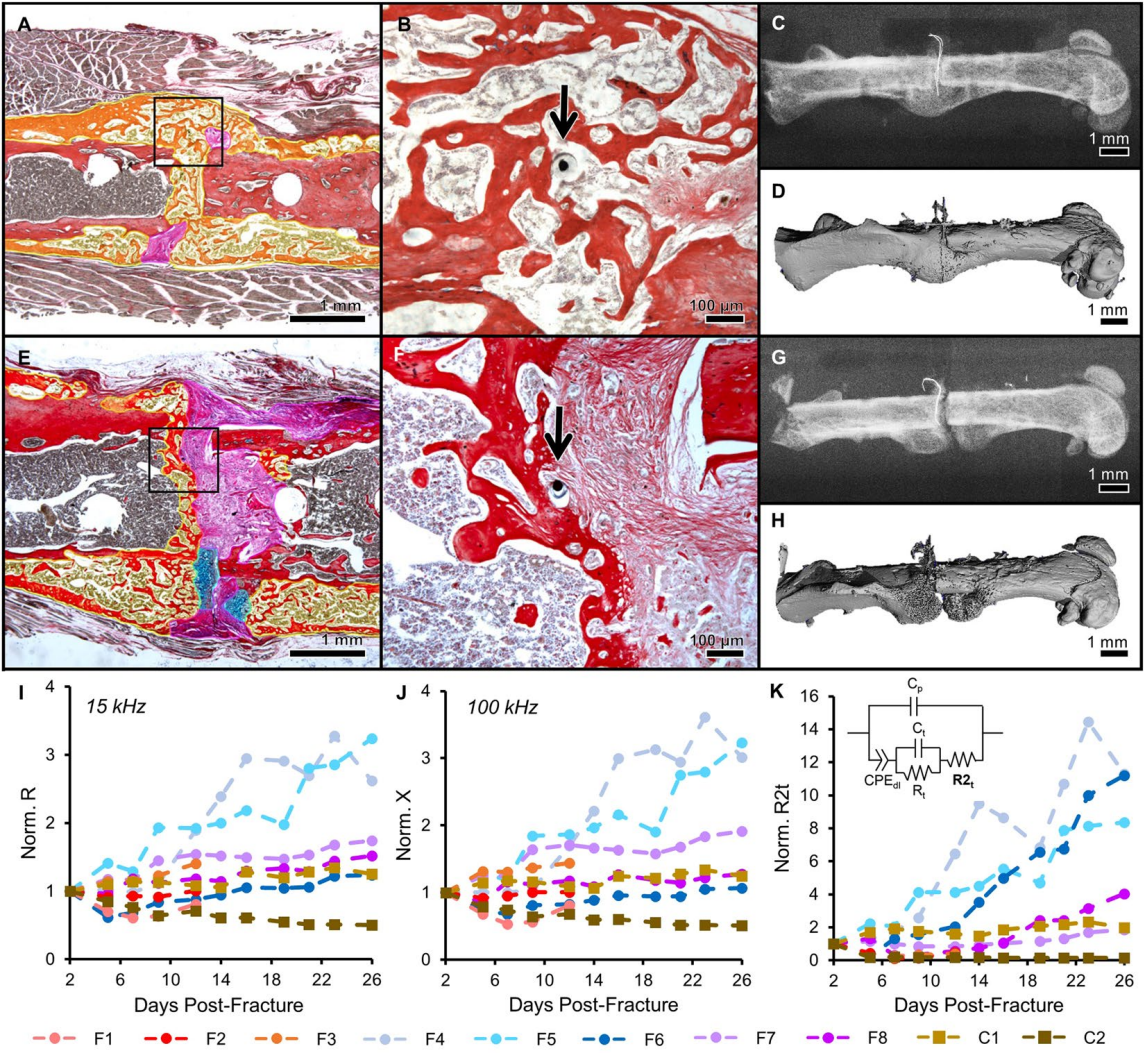
Impedance data distinguished femur fractures completely healed from those with varied healing in a bone plate model. Histology sections are stained and false-colored like in Fig. 2. (**A**) Representative histology section of a well-healed mouse at day 26; a large bony callus completely bridges the fracture ends. Black box outlines position of the high-magnification image in (**B**). (**B**) High-magnification image of (**A**) with black arrow pointing to the electrode fully-integrated in new trabecular bone. (**C**) X-ray radiograph of sample in (**A**). (**D**) Surface rendered, three-dimensional µCT image of sample in (**A**). (**E**) Representative histology section of a mouse with mixed healing at day 26; the fracture callus includes cartilage, fibrous tissue, and trabecular bone. Black box outlines position of the high-magnification image in (**F**). (**F**) High-magnification image of (**E**) with black arrow pointing to the electrode fully-embedded in the callus, surrounded by a mixture of new trabecular bone and fibrous tissue. (**G**) X-ray radiograph of sample in (**E**). (**H**) Surface rendered, three-dimensional µCT image of sample in (**E**). (**I**) R (normalized as a ratio to day 2) at 15 kHz plotted over days post-fracture. Data markers and lines are colored according to degree of healing – shades of red for mice sacrificed at day 12 (F1, F2, F3), shades of blue for mice sacrificed at day 26 that healed well (F4, F5, F6), shades of purple for mice sacrificed at day 26 that healed poorly (F7, F8), and shades of brown for control mice sacrificed at day 26 (C1, C2). Normalized R clearly rises at a faster rate in two mice with complete bony calli, F4 and F5. (**J**) X (normalized as a ratio to day 2) at 100 kHz plotted over days post-fracture. Normalized X clearly rises at a faster rate in two mice with complete bony calli, F4 and F5. (**K**) Impedance data at all measured frequencies is fit to an equivalent circuit model (inset), and the R2_t_ parameter is extracted, normalized as a ratio to day 2, and plotted over days post-fracture. This analysis is able to clearly distinguish the samples that are classified as union by orthopaedic surgeons (F4, F5, and F6 in Table 1).

**Figure 5:**
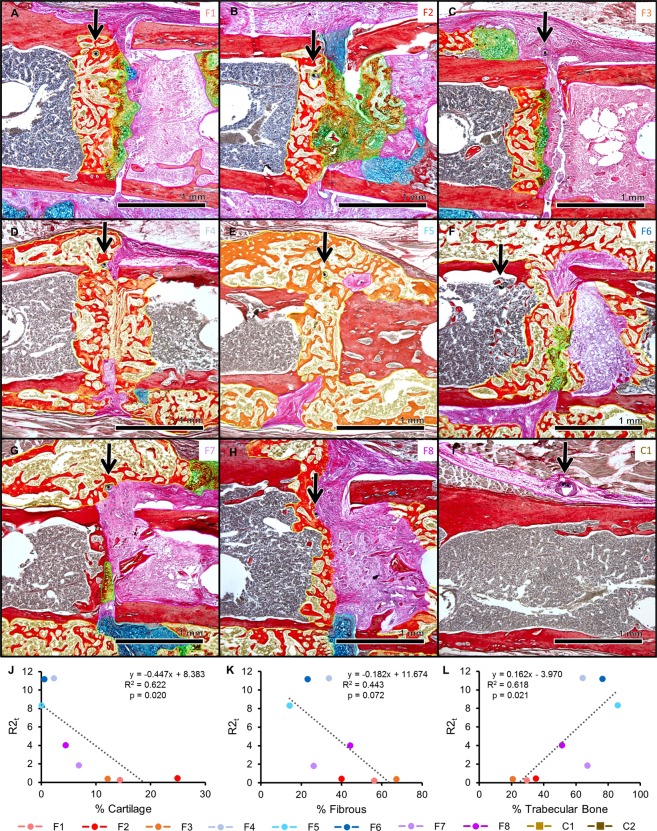
Histology sections for all bone plate model mice. Histology sections are stained and false-colored like in Fig. 2. Green = cartilage converting to bone. Black arrows point to sensors embedded in the fracture callus. Sample labels (upper right corner) are colored to match the graphs in Fig. 4I–K. (**A–C**) Histology sections for mice sacrificed at day 12. (**D–F**) Histology sections for mice sacrificed at day 26 with fracture calli composed almost entirely of new trabecular bone. (**G–H**) Histology sections of mice sacrificed at day 26 that experienced mixed healing. (**I**) Representative histology section of a control mouse, where the sensor is embedded in a small amount of fibrous tissue. (**J–L**) R2_t_ is significantly correlated to % cartilage as well as to % trabecular bone. R2_t_ also trends with % fibrous tissue. Individual markers are colored to match the corresponding samples in Fig. 4I–K.

As expected, the mice experienced varied degrees of healing. Some fractures healed very well, with complete bony bridging seen by histology, X-ray, and µCT (Figs 4A,C,D and S3A). While all samples showed evidence of a healing response, in some cases a bony callus did not bridge the fractured bone ends (Figs 4E,G,H and S3B). We were again able to define the exact tissues immediately surrounding the sensor in each sample (Fig. 4B,F), regardless of global callus composition. Histology (Figs 5 and S4), X-ray, and µCT images (Fig. S5) for each sample indicate resultant healing state. In the control mice, the sensors stayed between the unfractured bone and muscle, but were encapsulated by some fibrous tissue (Figs 5I and S6). Stereology techniques were used to quantify the amounts of cartilage, fibrous, new bone, and new marrow tissue in each callus, and fracture callus composition was calculated as a ratio to the total callus volume (Table S1). Since bone remodels in the last stage of healing and thus produces a nonlinear relationship over the course of healing, we looked to % cartilage as a more appropriate variable to identify thresholds for union/nonunion classification. Since the amount of cartilage in a callus peaks between days 6 to 10 in a mouse model^16^ and our samples were all collected on or after day 12, % cartilage is expected to drop off as it is replaced by bone. Thus, samples with % cartilage values below 3% were considered “union” (U), values above 10% were considered “nonunion” (NU), and values in between were considered “suspected nonunion” (SNU) (Table 1).

**Table 1:** Categorization by X-ray, µCT, stereology, and EIS.

Sample	Total RUST Score (Mean ± Std. Dev.)	Max - Min	Categorization (# of Surgeons)			Cat. by µCT	Cat. by Stereology	Cat. by EIS
			U	NU	SNU			
F1	4.8 ± 1.10	3	1	1	3	NU	NU	NU
F2	4.8 ± 1.10	3		2	3	NU	NU	NU
F3	6 ± 1	2		1	4	NU	NU	NU
F4	8.4 ± 0.55	1	5			U	U	U
F5	8.8 ± 0.84	2	5			U	U	U
F6	9 ± 0	0	5			SNU	U	U
F7	9 ± 0	0	2		3	SNU	SNU	NU
F8	7 ± 1	2	1	3	1	SNU	SNU	NU

Total RUST scores for each sample were averaged amongst five orthopaedic surgeons and presented as mean ± standard deviation. The maximum score subtracted by the minimum score for each sample was calculated to show the largest difference between surgeon evaluations. The number of surgeons who categorized each sample as “union” (U), “nonunion” (NU), or “suspected nonunion” (SNU) are also provided. In clearly healed cases (F4,F5,F6) there is strong agreement between clinicians, but results vary for ambiguous cases. Categorizations based on thresholding µCT, stereology, and EIS data are also presented.

Well-healed samples like F4 and F5 show sensors embedded in new bone and exhibit electrical resistance (R) and reactance (X) values that grow steadily over time (Fig. 4I,J). Conversely, samples like F7 and F8 have a mix of new bone and fibrous tissue that result in R and X values that do not rise as high, indicating stagnant healing. Measurements in control samples remained relatively flat over time. By examining normalized R and X values at specific frequencies over healing time, we can begin to discriminate between clearly healed samples and those with mixed healing.

### Model-driven fit of data robustly classifies healing trends

To determine how the data best tracked healing, we fit the resistance and reactance data at all measured frequencies to an equivalent circuit model of biological tissue. This enabled us to extract the dominant electrical properties that explain our observed differences in healing. The equivalent circuit (Fig. 4K) models the electrode-electrolyte interface as a constant phase element (CPE_dl_) in series with the tissue component, and places a capacitor (C_p_) in parallel to reflect the parasitic capacitance due to coupling along the two serpentine traces. The tissue component is comprised of a resistor (R_t_, the ionic intracellular environment) and capacitor (C_t_, the double-layer cell membranes) in parallel, in series with another resistor (R2_t_, the ionic extracellular environment). Model parameters are detailed in the Methods section, and goodness of fits graphs are provided in Fig. S7. We found that normalized R2_t_ (Fig. 4K) accurately recapitulates healing state, with the best healed samples (F4, F5, and F6) rising significantly higher than the samples with more varied healing (p < 0.002). A threshold was set at R2_t_ = 5, above which fractures were categorized as “union” (U), as noted in Table 1. To examine the relationship between EIS and tissue composition, the extracted R2_t_ parameter for every sample was compared with the corresponding percentages of cartilage, fibrous tissue, and trabecular bone (includes new bone and new marrow) found in each callus at euthanasia. Regression analyses reveal that R2_t_ is negatively correlated with % cartilage (R^2^ = 0.622, p = 0.02), has a negative trend with % fibrous tissue (R^2^ = 0.442, p = 0.072), and is positively correlated with % trabecular bone (R^2^ = 0.618, p = 0.021) (Fig. 5J–L).

### Impedance correlates with µCT measures of healing

To understand how clinicians would characterize healing using existing classification techniques, five orthopaedic surgeons scored the final X-rays for each sample using the modified Radiographic Union Scale for Tibial (RUST) fractures^46^, with a total score from 3 to 12. Each X-ray was also categorized as “union”, “nonunion”, or “suspected nonunion” (Tables 1 and S2). In clearly healed cases like F4, F5, and F6, there is strong agreement between surgeons in total RUST score and all categorized these as fully united. This corroborates our impedance data, as the R2_t_ parameter extracted from the model-driven fit also classified these three samples as well-healed. However, surgeon evaluation varied for ambiguous cases, with a wider spread in total RUST score (up to 3 points difference) as well as disagreement in categorization, substantiating the lack of a standard for assessing fracture healing by X-ray.

**Figure 6:**
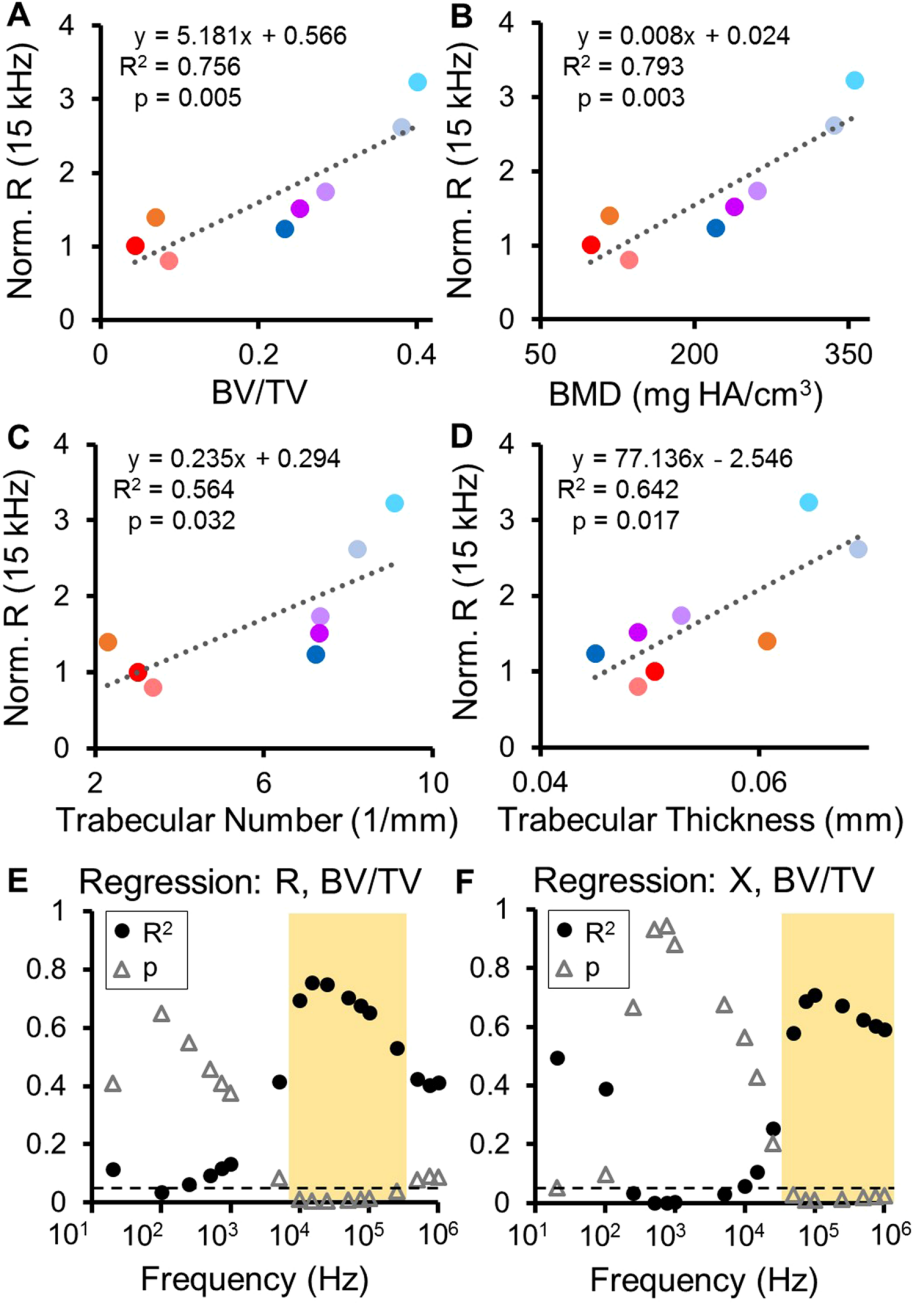
Regression analyses comparing normalized impedance data to µCT indices. (**A–D**) Normalized R at 15 kHz is significantly correlated to the ratio of bone volume to total volume (BV/TV), bone mineral density (BMD), trabecular number, and trabecular thickness. Individual markers are colored to match the corresponding samples in Fig. 4I–K. (**E**,**F**) Resultant R^2^ and p-values from regression analyses between normalized R and BV/TV as well as normalized X and BV/TV plotted as a function of frequency. Significance is set at p < 0.05 (below dashed line). Significant relationships (highlighted in yellow) between R and BV/TV occur from 10 kHz to 250 kHz, and significant relationships between X and BV/TV occur from 50 kHz to 1 MHz.

In the clinic, computed tomography (CT) scans can be requested to confirm or reject suspected delayed or nonunion cases. While CT sees limited use due to high costs, it does enable quantitative measures that reflect biological and structural measures of mineralized bone. We quantified a number of microstructural indices from µCT scans of our samples to determine their degree of correlation with EIS, including bone volume (BV), total volume (TV), bone mineral density (BMD), trabecular number, trabecular thickness, and trabecular separation (Table S3). We selected BMD to determine thresholds for union/nonunion classification because this index should be monotonic over healing in the time frame of our study^47^. Samples with BMD values above 300 were considered “union”, values below 200 were considered “nonunion”, and values in between were labeled “suspected nonunion” (Table 1). Following linear regression analyses, we find that normalized resistance (R) significantly correlates with the ratio of bone volume to total volume (BV/TV), BMD, trabecular number, and trabecular thickness (Fig. 6A–D). These correlations peak at 15 kHz, with normalized R rising with increasing BV/TV (R^2^ = 0.756, p = 0.005) and BMD (R^2^ = 0.793, p = 0.003). In addition, normalized R at 15 kHz has a positive relationship with trabecular number (R^2^ = 0.564, p = 0.032) and trabecular thickness (R^2^ = 0.642, p = 0.017), but no correlation with trabecular separation (R^2^ = 0.3599, p = 0.116). Similar relationships are found by comparing normalized reactance (X) to these µCT indices.

Importantly, by plotting the associated R^2^ and p values at every measured frequency for each pair of variables, we can pinpoint the frequency ranges in which impedance provides valuable information about healing. Normalized R is significantly correlated to BV/TV from 10 kHz to 250 kHz (R^2^ > 0.5, p ≤ 0.04), with a peak at 15 kHz (Fig. 6E). Normalized X is significantly correlated to BV/TV from 50 kHz to 1 MHz (R^2^ > 0.55, p < 0.03), with a peak at 100 kHz (Fig. 6F). Analogous graphs for the other µCT indices are shown in Fig. S8, with results summarized in Table S4. These regression analyses not only determine that EIS correlates to clinically-relevant measures of bone healing that would otherwise require expensive µCT evaluation, but also identify the specific frequency ranges at which this is most significant.

## Discussion

Our study aimed to demonstrate the use of EIS to develop smart fracture fixation instrumentation, with the goal of arming physicians with quantitative information about the state of fracture repair in patients. While previous research has utilized impedance to evaluate bone fractures, other groups have used surface electrodes outside the leg or pins drilled in the proximal and distal ends of the bone. As a result, EIS measurements in these studies reflect global assessments with low specificity to the changing fracture callus, as signal must also travel through surrounding muscle and skin. To our knowledge, our study is the first to design and fabricate microscale sensors implanted directly in the fracture gap intra-operatively. This enabled longitudinal EIS measurements to be taken specifically in the calli of individual mice with fractures stabilized using either external fixators or bone plates *in vivo*.

Taken together, the results show that EIS tracks differences between fractures healing well and those healing poorly in two different murine models, laying the foundation for use of this technique to detect fracture nonunion or other complications. Using small, flexible sensors, we measured EIS in fractures producing a range of healing responses from atrophic nonunion to full bridging. These were also assessed with a comprehensive range of standard clinical and laboratory techniques including histology, X-ray with assigned RUST scores, and µCT. Due to the small size of our sensors, histology could confirm not only the tissue composition across the entire fracture gap, but also identify the exact tissue the sensor was embedded in. While sensor motion in some samples likely contributed to excess fibrosis, histological evidence showed that our microscale electrodes were well integrated within fracture tissue and did not prevent complete bony bridging when appropriately immobilized. Full integration of our sensors within fracture calli was critical for taking local measurements of healing tissue over time in individual mice without disturbing the repair process.

We found that impedance between mice that healed well and those that healed poorly deviated quickly, with electrical resistance and reactance rising at a faster rate in well-healed fractures. This indicates that impedance may be able to rapidly identify stalled healing as compared to a traditional technique such as X-ray, which can only detect mineralized tissue at late stages. Resistance (R) reflects the ability of a material to conduct charge – a highly conductive material will exhibit a small resistance, while a less conductive material will have a larger resistance. As the dominant tissue in a fracture callus transitions from highly conductive blood and cartilage to less conductive trabecular and cortical bone^34^, our sensors confirm that R rises sharply over time in mice that exhibited a clear healing response. Reactance (X) describes effects that store energy such as charge separation across cell membranes (which act as barriers to charge flow and look capacitive). Highly cellular tissues have a higher capacity for energy storage and thus a larger X, particularly at higher frequencies. Because bone consists of capacitive matrix layers and marrow is highly cellular, we see an acute rise in X in mice with bridging calli, in contrast to moderate changes in mice with poor healing. Both R and X stayed roughly flat in control mice (no fracture), with minor deviations due to small fibrotic responses. We also find the greatest spread in R and X across days from 1 kHz to 100 kHz, with formation of a robust callus influencing frequency response of the measurement. Spectroscopic measurements thus enable us to precisely track where changes arise in the frequency range over healing time. These results align with previous work in cadaveric and *ex vivo* mouse models^33,34^ that describe resistive and reactive components increasing over fracture healing time (from blood to cartilage to bone). EIS data collected in both the external fixator and bone plate models signify consistency of this technique in two different fracture fixation schemes.

By fitting our data to an equivalent circuit model, we were able to combine the effects of resistance and reactance changes at all measured frequencies and determine robust measures of trends over time. By simplifying the complex physical components present in healing fractures to electrical components that suitably approximate tissue^24,48^, our analysis found that the R2_t_ term describing extracellular environment clearly separated the mice with bony bridging from those without. Although the model is an approximation of the biology occurring between the two electrodes in the fracture gap, it allowed us to employ all the impedance data in a combined fashion to help explain the differences in healing that we observed. With validation from a larger clinical trial, this model could serve as a platform to create prognostic tools in the clinic to predict and differentiate proper healing and delayed or nonunion.

In mice with clearly healed fractures, all techniques showed strong agreement in classifying the fractures as united. However, in more heterogeneous cases, there was no consensus among the five surgeons. Notably, there were two cases that were categorized as “union”, “nonunion”, and “suspected nonunion” depending on the surgeon being asked, where classification by EIS matched the majority of surgeon opinion. This further emphasizes the need for an objective method to quantify the degree of fracture repair, as interpretation of X-rays can vary amongst surgeons, potentially due to factors like medical training, experience, etc. In order to correlate EIS data to more accurate measures of healing, we derived quantitative data from µCT. Our regression analyses show that both normalized R and X have strong positive relationships with BV/TV, BMD, trabecular number, and trabecular thickness within specific frequency ranges. These correlations peak in significance at 15 kHz for R and at 100 kHz for X, reflecting the primary frequencies of interest to understand mineralization of the fracture callus. Mineralized bone as a ratio of the total callus volume as well as bone mineral density rise as chondrocytes become mineralized and then convert to bone. Likewise, as fractures transition from a solid cartilage callus to trabecular bone, we expect trabecular number and thickness to increase. Our impedance measurements are able to track these changes with a high degree of sensitivity, and with future clinical studies, may be able to provide enough extra information to a physician to increase their confidence in a diagnosis without requiring the extra expense and radiation exposure associated with CT scans. To determine tissue composition on a cellular level, we used stereology techniques to quantify histological images. We found that the R2_t_ parameter extracted from our EIS data correlates with % cartilage and % trabecular bone as expected, with this resistive term decreasing with larger quantities of cartilage (more conductive) and increasing with larger quantities of trabecular bone (less conductive). By studying the categorizations of “union”, “nonunion”, and “suspected nonunion” based on three additional methods of monitoring, we can examine how EIS compares to traditional techniques. While X-ray interpretation was subjective and led to a number of discrepancies between surgeons, µCT provided quantitative data from which we could create objective thresholds. Categorizations by stereology were consistent with the majority opinion of surgeons, but while histology remains the gold standard for assessing extent of repair, it cannot be conducted in a clinical setting. Categorizations by EIS were closely matched to those made by stereology, underscoring the utility of this technology to reflect tissue composition *in vivo*.

While mice provide a well-established model of fracture healing that closely mimics the same process in humans, a major challenge was designing wired implants within tight size constraints. Multiple redesigns were necessary to prevent mice from chewing or otherwise damaging their sensors, but difficulties in collecting data over the entire healing time led to the removal of data from some mice whose sensors broke. While small sensors improved integration in fracture tissue, they were prone to movement and had higher impedance, possibly reducing the signal-to-noise ratio of our measurements. Future designs could use stiffer electrodes and increase electrode surface area to limit this effect, and focus on achieving high animal numbers in a larger animal model. By integrating wireless data transfer, a sensor could be added intra-operatively as a simple addition to standard orthopaedic hardware. For example, electrodes could be incorporated into an instrumented bone screw used with a standard plate. Following fixation, a surgeon would insert this “sensor screw” through a free bone plate hole above the fracture. The fracture would heal around the screw while the sensor provided data, and the plate could be left implanted as per usual practice. This would provide detailed information to physicians and patients about healing trends at much higher temporal resolution than currently available via conventional follow-up appointments spaced weeks apart. Furthermore, EIS holds promise for early detection of fracture nonunion, which currently can take over 6 months to diagnose, as it is more sensitive to early stages of hematoma and cartilage than traditional techniques.

In this paper, we have presented complete histologic, radiographic, and µCT analyses with our data to show that local EIS measurements can evaluate fracture calli with high sensitivity. Use of both external fixators and bone plates demonstrates the value of EIS in different models with different rates and progression of healing, with even the smallest fixation implants providing convenient platforms for adding sensors and instrumentation. This establishes EIS as a technology amenable to miniaturization that can be translated and easily integrated into current fracture management strategies in the clinic. Our study lays the groundwork for instrumented implants, which could be used during surgical intervention to provide physicians with additional quantitative information about the state of fracture repair during post-operative monitoring and help guide clinical care decisions.

## Materials and Methods

### Study design

Our goal for this study was to assess the feasibility of EIS as a quantitative tool to monitor fracture healing. From previous studies, we hypothesized that EIS measurements would correlate well with biological indicators of healing, such as callus composition, X-ray radiographs, and µCT indices (i.e. bone mineral density). We conducted a controlled laboratory experiment in a murine fracture model, stabilizing fractures with an external fixator or bone plate and inserting custom sensors in the fracture gap. Euthanasia time points were selected to provide information about callus composition over the course of healing.

### Impedance measurement system

Two-point impedance measurements were taken at the following frequencies (Hz): 20, 100, 1 k, 5 k, 10 k, 15 k, 25 k, 50 k, 75 k, 100 k, 250 k, 500 k, 750 k, 1 M. Impedance magnitude (|Z|) and phase (θ) were recorded from an Agilent E4980AL Precision LCR Meter with a 100 mV sine wave output signal, and converted to electrical resistance (R) and reactance (X). 100 mV was chosen to be below the threshold for electrolysis of water, and as high as necessary to obtain good signal-to-noise ratio. At each measurement time point, data was averaged over three sets of measurements taken for each sample, with each set involving 5 impedance measurements at the 14 frequencies listed above.

### Sensor development

#### External fixator model

250 µm diameter sensor pins were fabricated using an FR4 (a glass-reinforced epoxy laminate material) substrate with electroless nickel immersion gold (ENIG) plated electrodes (127 µm diameter) spaced 0.5 mm apart (PCB Universe, Vancouver, WA). Smaller sensors were then made for a subsequent study to minimize disruption of healing. 25.4 µm diameter platinum (Pt) wires insulated with 1.27 µm isonel (A-M Systems, Sequim, WA) were used to create sensors small enough for implantation in a fracture model involving the mouse tibia, which is approximately 1 mm in diameter. Two wires were cut to roughly 50 mm in length, and recording sites were exposed by burning off short 175 µm sections of insulation in the center of each wire using a CO_2_ laser. The two wires were offset so the electrodes were spaced 0.5 mm apart, then twisted together to form a single sensor. The wires were coiled on either side of the electrodes to allow for strain relief.

#### Bone plate model

Flexible sensors were fabricated on 115 µm thick polyimide (AltaFlex, Santa Clara, CA) to be affixed to the bone plate. Epo-Tek H20E silver (Ag) epoxy (Epoxy Technology, Inc., Billerica, MA) was used to conductively adhere 25.4 µm diameter Pt wires to ENIG-plated pads on the polyimide sensor board. These Pt wires were attached at the center of the bone plate to serve as electrodes directly in the fracture gap. A long serpentine cable extended from the proximal end of the plate, leading to a set of connection pads that interfaced with our measurement instrumentation. The entire sensor was then coated with 6–15 µm of Parylene-C at room temperature through a chemical vapor deposition process using an SCS Labcoter® 2 Parylene Deposition System (Specialty Coating Systems, Indianapolis, IN) as an insulator and for biocompatibility. A sharp razor blade was used to cut off the ends of the Pt wires to expose recording sites (700 µm^2^) spaced 0.5 mm. Medical-grade Epo-Tek 353ND epoxy was used to attach the two Pt wires and to secure the polyimide board to the proximal half of the bone plate.

Prior to implantation, fixation instrumentation was autoclaved and sensors were sterilized by immersing in 70% EtOH under ultraviolet light for at least 1 hour.

### Mouse models

Approval was obtained from the University of California, San Francisco (UCSF) Institutional Animal Care and Use Committee (IACUC) prior to performing these mouse studies, the methods were carried out in accordance with the relevant guidelines and regulations, and this report adheres to ARRIVE Guidelines for reporting animal research^49^. Rodent models of fracture repair have been used since 1948^50^ and are well-established as preclinical models that provide insight into human fracture healing^51^. Wild-type C57BL/6J adult mice (male, 22–33 g, 12–20 weeks old; The Jackson Laboratory, Bar Harbor, ME) were housed in cages (≤5 mice/cage) with paper/cellulose bedding and easy access to food and water, and were monitored for signs of stress (low activity and unkempt fur from visual inspection, weight loss from scale measurements) at least three times per week. Mice aged 12–20 weeks are all considered mature adults^52^, with no differences in healing expected within this range. For both models below, mice were anesthetized with an intraperitoneal (IP) injection of ketamine:dexmedetomidine (1:1) for surgery, and revived with an IP injection of atipamezole. For pain relief, buprenorphine was injected subcutaneously immediately following surgery, and subsequently at four and twenty four hours post-surgery per the IACUC protocol. Mice were given a prophylactic dose of enrofloxacin antibiotic following surgery and an additional dose on the subsequent two days.

#### External fixator model

Fractures were made in the mid-diaphyses of mice and stabilized using custom-designed external fixators. The healing timeline for this model has been previously examined in detail^53^. Sensors were carefully placed in the fracture gap, with skin sutured closed around the ends of the sensors, leaving wire ends available for connection to measurement instrumentation. Mice were anesthetized twice weekly using isoflurane mixed with oxygen to track healing progression with impedance measurements on days 0, 4, 7, 11, 14, 18, 21, 25, and 28. A thin, rigid acrylic sheath was placed over the external fixator to protect the sensor in between measurements. Eleven mice were implanted with 250 µm sensor pins in two different models of post-injury tissue changes – a 2 mm defect was created by an osteotomy (N = 5) and a 0.5 mm defect was created by slight distraction of the proximal and distal bone ends (N = 6). A 1 V sine wave output signal was used for this study. Mice were euthanized at days 3 (N = 1), 4 (N = 1), 7 (N = 3), 10 (N = 1), 14 (N = 2), 25 (N = 1), and 28 (N = 2) for histology to reflect varying degrees of healing. Six mice were implanted with thin 56 µm Pt wire sensors in a 0.5 mm defect; 1 mouse was found dead on day 4 and taken out of the study. Mice were euthanized at day 28 for histology to allow for healing to progress to a late enough time point to diagnose proper healing or lack of healing.

#### Bone plate model

Ten mice had their right femurs stabilized with Titanium bone plates (RISystem, Davos, Switzerland), and defects (<0.25 mm wide) were made with a single cut of a Gigly saw in the mid-diaphysis. The progression of healing with this model has been previously examined in detail^54,55^. Two 700 µm^2^ electrodes spaced 0.5 mm apart were centered in the fracture gap. Surgery was performed on eight additional mice, placing a bone plate and sensor but without making a fracture, to serve as controls. In these mice, the two electrodes were placed beside the bone and the surrounding muscle was sutured over it. While infection was not a major issue, the mice had a tendency to chew or otherwise damage the connector outside the animal. To prevent this, mice were singly housed and a stretchable serpentine cable was tunneled subcutaneously up the back of each mouse, with a small incision made between the shoulder blades as an exit point. Connection pads at this location allowed for connection to measurement instrumentation. The muscle and skin was then sutured closed over the stabilized fracture. To monitor the progression of healing, mice were anesthetized with isoflurane mixed with oxygen three times each week for measurements on days 0, 2, 5, 7, 9, 12, 14, 16, 19, 21, 23, and 26. Mice were euthanized at day 12 (N = 4) and day 26 (N = 6 fractured, N = 8 control) for histology and µCT; these two time points were chosen to reflect the transition from stage 2–3 (day 12 – mixture of cartilage and trabecular bone) and stage 3–4 (day 26 – dominated by trabecular bone, beginning to remodel). 8 mice were taken out of the study (N = 1 fracture at day 12, N = 1 fracture at day 26, N = 6 control at day 26) because their sensors broke at the serpentine junction, as seen in radiographic images and confirmed during dissection, possibly due to excessive movement by the animal.

In both models, sensors were placed centrally in the fracture gaps, with two electrodes spaced 0.5 mm apart. Each electrode was placed just inside of opposing cortices in order for EIS measurements to capture the tissue spanning the entire thickness of the fractured bone. Signal from the electrodes travels radially, but current also preferentially moves from one electrode to the other and through paths of lower impedance. The experimental study design is summarized in Table S5.

### Radiography

Fluoroscopy images were taken using a Hologic Fluoroscan Premier Encore 60000 C-Arm Imaging System at every measurement time point. Following euthanasia, the femur specimens were harvested and fixed overnight before being transferred to 70% ethanol for scanning. X-ray radiography was performed using a Scanco µCT 50 high-resolution specimen scanner (Scanco Medical AG, Bassersdorf, Switzerland). Specimens were imaged with X-ray energy set to 55 kVp and 109 µA. Orthogonal images were taken first with the plate intact and then again after removing the plate and screws. Orthogonal images of the bones with the plate removed were scored by five orthopaedic surgeons (single-blind assessment) using the modified Radiographic Union Scale in Tibia (RUST) fractures, with each cortex being assigned a number from 1 to 4 (1 = no callus, 2 = callus present, 3 = bridging callus, 4 = remodeled with no visible fracture line)^46^. The anterior cortex was excluded as the sensor typically occluded its view on an X-ray, so total values for each specimen could range from 3 to 12 by adding up the scores on the remaining three cortices. Surgeons were also asked to clinically diagnose the fractures as “union” (U), “nonunion” (NU), or “suspected nonunion” (SNU).

Microcomputed X-ray tomography (µCT) was performed with the entire specimen scanned at an isotropic resolution of 10 µm, with an X-ray tube potential of 55 kVp and X-ray intensity of 109 µA. Integration time was set at 500 milliseconds per projection. After scanning, 3D microstructural image data was reconstructed using Scanco cone-beam reconstruction algorithm. Density calibration of the scanner was performed using a hydroxyapatite calibration phantom provided by the manufacturer. Structural indices were calculated using Scanco evaluation software (version 6.0; Scanco Medical AG). Using this software, the fracture callus was manually delineated from surrounding tissue at its cortical surface and bone morphology was assessed in the region between the central surgical screws (single-blind assessment). The callus tissue was isolated from preexisting bone by performing manual delineation to exclude the original diaphyseal cortex and its intramedullary space. The bone volume was segmented with a lower threshold of 240 per-mille grayscale units (grayscale values range from 0 to 1000) and image noise was reduced using a Gaussian filter set at a sigma and support of 0.8 and 1, respectively. Three-dimensional microstructural indices reported include bone volume (BV), total volume (TV), bone mineral density (BMD), trabecular number, trabecular thickness, and trabecular separation. Surface rendered, three-dimensional images were generated using Scanco Ray v4.0 software.

### Histology

Samples were fixed immediately after dissection in 4% paraformaldehyde (pH 7.2) overnight at 4 °C, then decalcified in Cal-Ex (Fisher Scientific, Hampton, NH) for 48 hours at room temperature after completion of µCT scans. Samples were then processed through serial ethanol dilutions and xylene dehydration, embedded in paraffin, and serial 10 µm sections were collected and stained with Hall’s and Brunt’s Quadruple (HBQ) stain, which stains cartilage tissue blue and bone red.

Callus compositions of femur samples were quantified between the central surgical screws using unbiased and randomized stereology principles^56–58^ on an Olympus BX51 microscope and CAST stereology system (Olympus Corp., Tokyo, Japan) with Visiopharm Integrator System (Visiopharm A/S, Hoersholm, Denmark) software. Cartilage, fibrous tissue, new bone, and new marrow were identified by randomly sampling over a grid of 36 evenly spaced points at 20x magnification over 30% of the tissue, with total tissue volumes calculated using Cavalieri’s principle. Six sections spaced 300 µm were analyzed for each sample, and the original diaphyseal cortex and its intramedullary space were excluded. New bone and new marrow were combined in regression analyses to reflect the trabecular bone false-colored in yellow in the histology images. Composition measurements were calculated as each fracture tissue volume divided by total callus volume.

### Equivalent circuit model

The equivalent circuit models the double-layer impedance at the electrode-electrolyte surface as a constant phase element (CPE_dl_) in series with the tissue component, and places a capacitor (C_p_) in parallel to reflect the parasitic capacitance due to coupling along the two long wire traces leading from the electrodes to the connector. The tissue component is comprised of a resistor (R_t_) and capacitor (C_t_) in parallel, in series with another resistor (R2_t_). R_t_ represents the ionic intracellular environment, R2_t_ represents the ionic extracellular environment, and C_t_ represents the double layer cell membranes. In our model, we held C_p_ constant at 25 µF, extracted from high-frequency measurements taken with our sensors immersed in Phosphate Buffered Saline (PBS) prior to implantation. The impedance of CPE_dl_ is calculated as

2 $${{\rm{Z}}}_{{\rm{CPE}}}=1/{\rm{Q}}{(j{\rm{\omega }})}^{{\rm{\alpha }}}$$

where Q is the numerical value of admittance (1/|Z|) at ω = 1 rad/s, ω is the angular frequency, and α is a constant from 0 to 1 (1 representing a perfect capacitor). We held α constant at 0.5, based on low-frequency measurements of our sensors in PBS. From a study characterizing an osseous implant using EIS^48^, we hypothesized that the changing impedance in our study would be most reflected in R2_t_. We thus fit R_t_ and C_t_ at an initial time point (day 2) and constrained it at that value over time. We used a custom MATLAB script to fit our data at each time point to this model, interpolating 10 steps in between each time point to guide the mathematical solution and avoid jumping to non-physical values. Extracted R2_t_ values were normalized as a ratio to day 2.

### Statistical Analysis

Longitudinal impedance data was normalized to the 2^nd^ measurement time point to allow the injury to stabilize. Univariate linear regression analyses were performed to compare end-point impedance measurements to stereology results (quantified tissue composition) and to a number of µCT indices (bone volume/total volume, bone mineral density, trabecular number, trabecular thickness, and trabecular separation). Two-tailed *t*-tests determined whether regression slopes were significantly different than zero, and significance was set at p < 0.05.

## Supplementary information

Smart bone plates can monitor fracture healing - Supplementary Materials

## Data Availability

All data needed to evaluate the conclusions are present in the paper and/or the Supplementary Materials. Additional information related to this paper may be requested from the authors.
