# Supplementary material for: Smart bone plates can monitor fracture healing
Source: Sci Rep. 2019 Feb 14;9:2122. doi: 10.1038/s41598-018-37784-0 (PMC6375940; doi:10.1038/s41598-018-37784-0)
Supplement: Supplementary file 1 — Smart bone plates can monitor fracture healing - Supplementary Materials [file 41598_2018_37784_MOESM1_ESM.pdf]

Smart bone plates can monitor fracture healing

Authors: Monica C. Lin[1]*, Diane Hu[2], Meir Marmor[2], Safa T. Herfat[2], Chelsea S. Bahney[1,2,3†], Michel M. Maharbiz[1,4,5†]

Affiliations:
[1]Department of Bioengineering, University of California, Berkeley, CA 94720, USA.
[2]UCSF Orthopaedic Trauma Institute, Zuckerberg San Francisco General Hospital, San Francisco, CA 94110, USA.
[3]Center for Regenerative Sports Medicine, Steadman Philippon Research Institute, Vail, CO, 81657 USA
[4]Department of Electrical Engineering and Computer Science, University of California, Berkeley, CA 94720, USA.
[5]Chan Zuckerberg Biohub, San Francisco, CA 94158, USA.
†Co-senior author.
*Corresponding author: monica.lin@berkeley.edu

Supplementary Materials

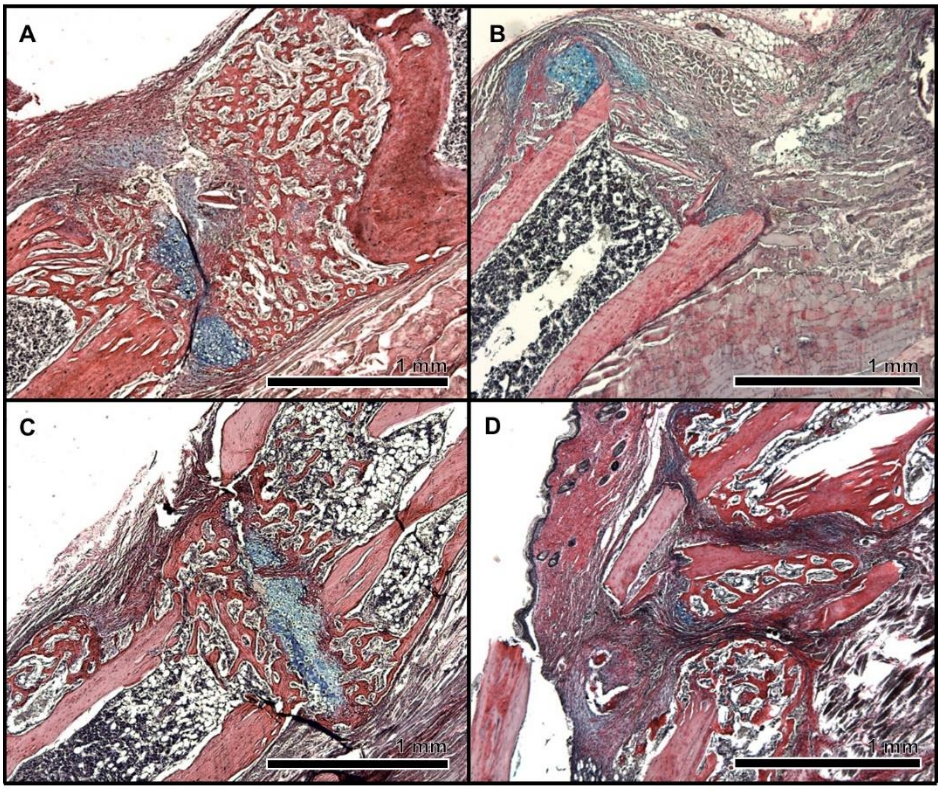

Figure S1 – Original histology images of external fixator model samples (Fig. 2A-B,D-E).
Histology sections are stained with HBQ (blue = cartilage, red = bone). **(A)** Original image of the histology section in Fig. 2A of an externally-fixed 0.5 mm defect at 14 days post-fracture. **(B)** Original image of the histology section in Fig. 2B of an externally-fixed 2 mm defect at 14 days post-fracture. **(C)** Original image of the histology section in Fig. 2D of an externally-fixed well-healed mouse at day 28. **(D)** Original image of the histology section in Fig. 2E of an externally-fixed poor-healed mouse at day 28.

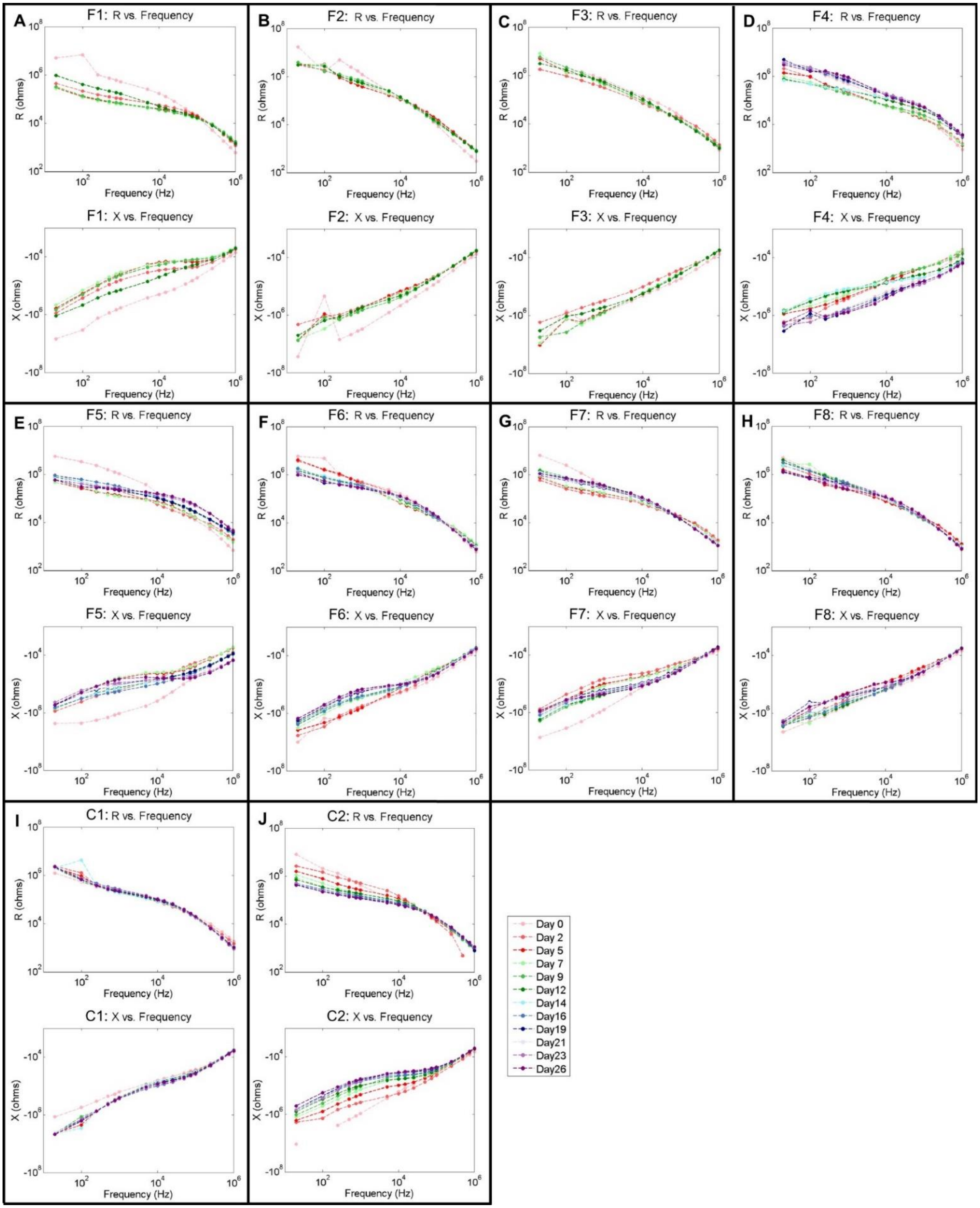

Figure S2 – Frequency response over time in all bone plate model mice.

(A-C) Electrical resistance (R) and reactance (X) plotted as a function of frequency for each measurement day in mice sacrificed on day 12. **(D-F)** R and X plotted as a function of frequency for each measurement day in mice sacrificed on day 26 with calli composed nearly completely of new trabecular bone. **(G-H)** R and X plotted as a function of frequency for each measurement day in mice sacrificed on day 26 that experienced mixed healing. **(I-J)** R and X plotted as a function of frequency for each measurement day for control mice.

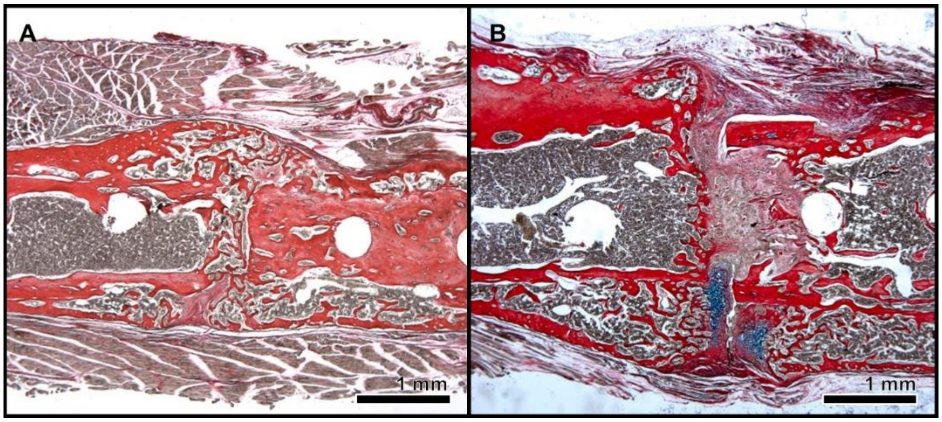

Figure S3 – Original histology images of well-healed and mixed-healed bone plate model samples (Fig. 4A,E). Histology sections are stained with HBQ (blue = cartilage, red = bone). **(A)** Original image of the histology section in Fig. 4A of a fracture fixed with a bone plate at 26 days post-fracture that was well-healed. **(B)** Original image of the histology section in Fig. 4E of a fracture fixed with a bone plate at 26 days post-fracture that had a mixed healing response.

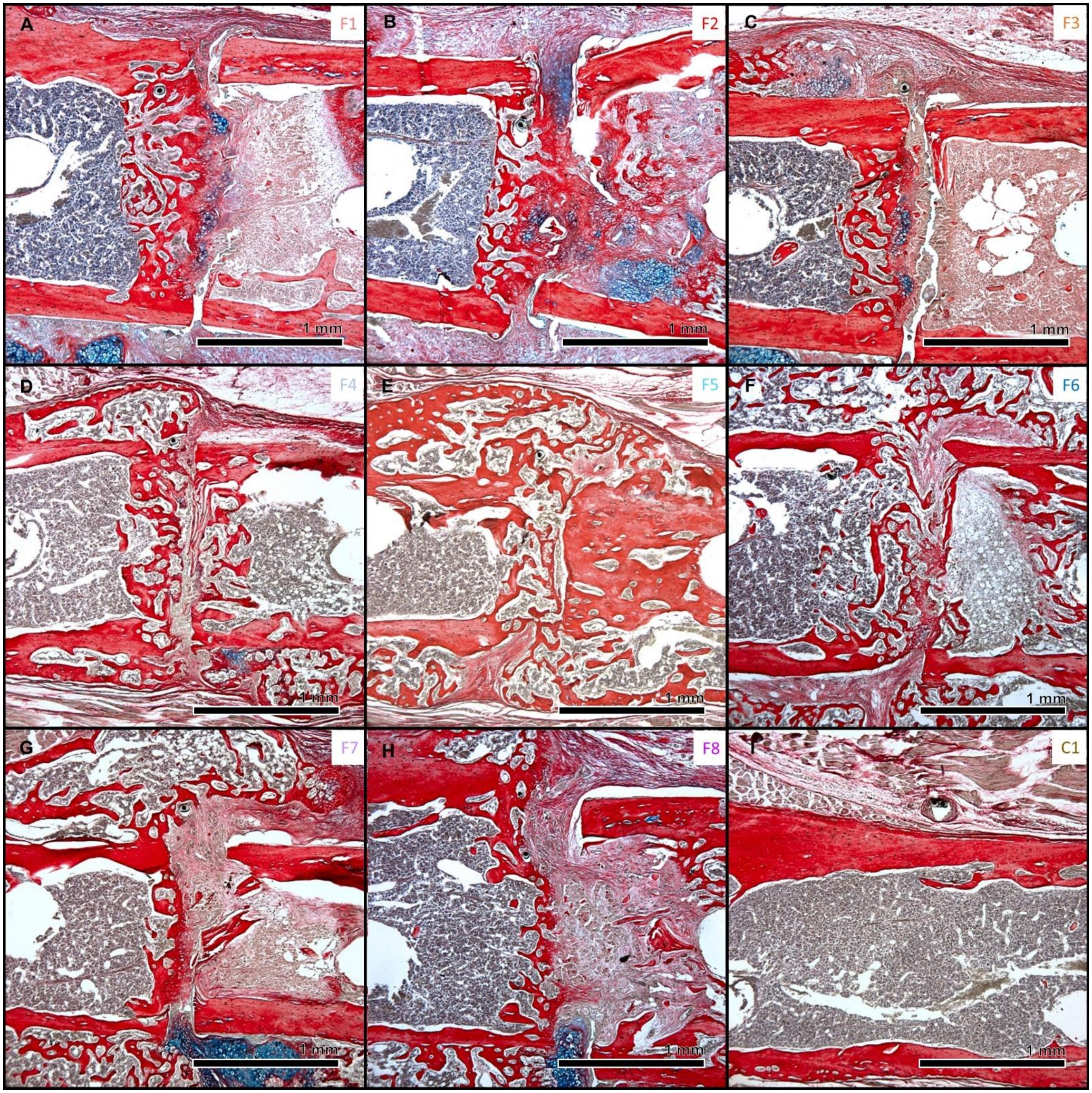

Figure S4 – Original histology images of all bone plate model samples (Fig. 5A-I).
Histology sections in this figure are stained with HBQ (blue = cartilage, red = bone). **(A-C)** Original images of the histology sections in Fig. 5A-C of mice sacrificed on day 12. **(D-F)** Original images of the histology sections in Fig. 5D-F of mice sacrificed on day 26 with calli composed nearly completely of new trabecular bone. **(G-H)** Original images of the histology sections in Fig. 5G-H of mice sacrificed on day 26 that experienced mixed healing. **(I)** Original image of the histology section in Fig. 5I of a control mouse on day 26.

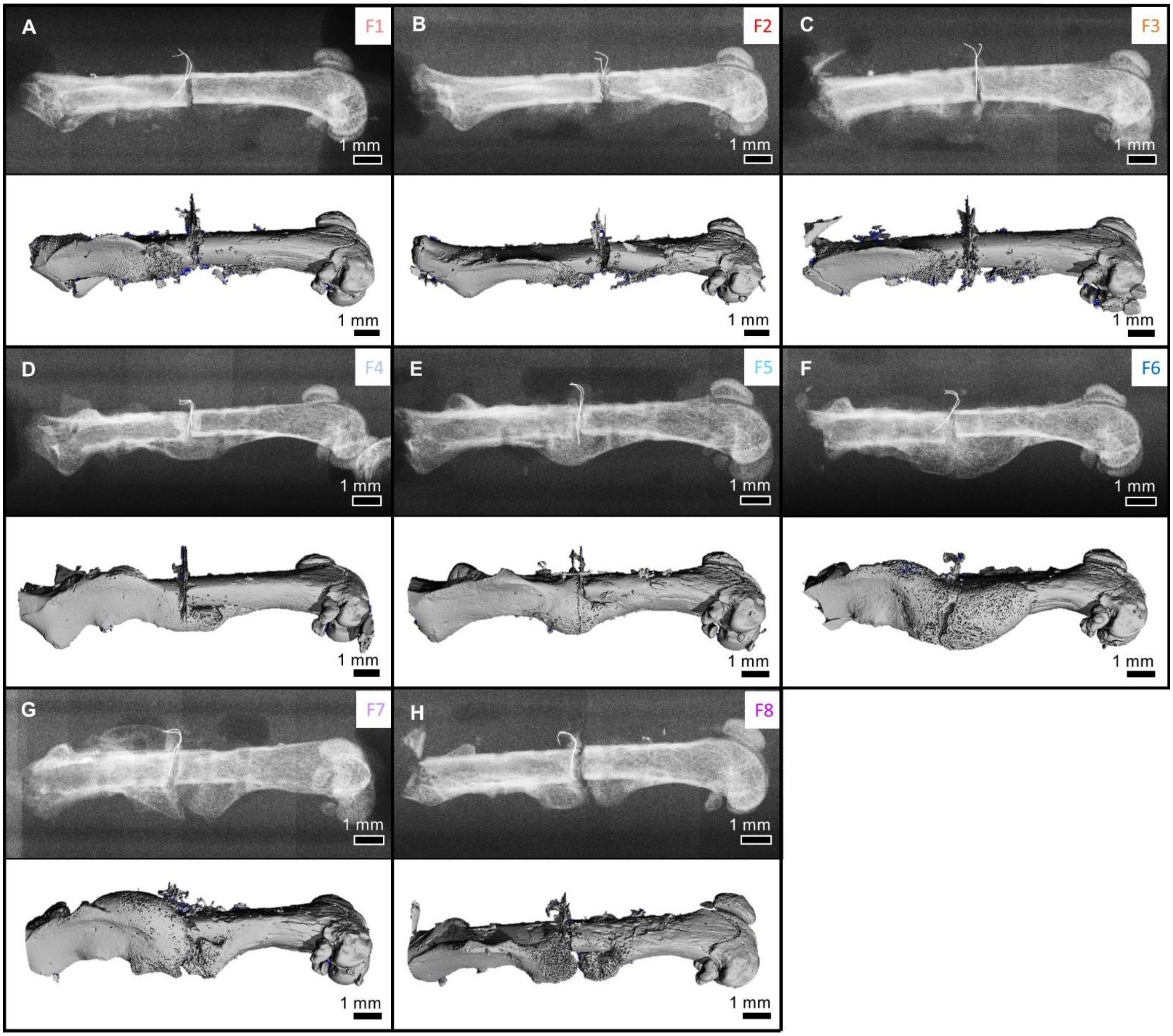

Figure S5 – X-ray and µCT images of all bone plate model fracture samples.
(A-C) X-ray and µCT images of femurs from mice sacrificed on day 12. **(D-F)** X-ray and µCT images of femurs from mice sacrificed on day 26 with calli composed nearly completely of new trabecular bone. **(G-H)** X-ray and µCT images of femurs from mice sacrificed on day 26 that experienced mixed healing.

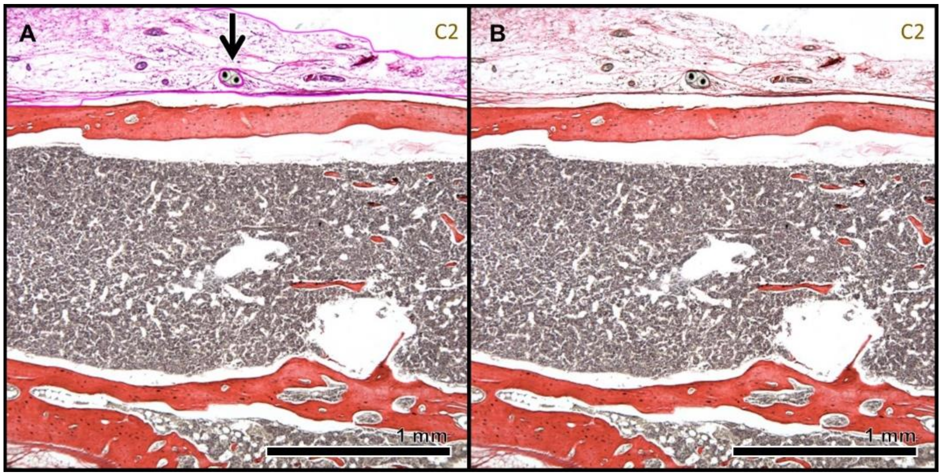

Figure S6 – Histology image for control mouse.

Histology sections in this figure are stained with HBQ (blue = cartilage, red = bone). **(A)** Histology section is false-colored to aid interpretation of tissue composition. Purple = fibrous/amorphous tissue. Original red color = cortical bone, black/white area = bone marrow. Black arrow points to sensors embedded in fibrous tissue next to the unfractured bone. **(B)** Original histology section from (A).

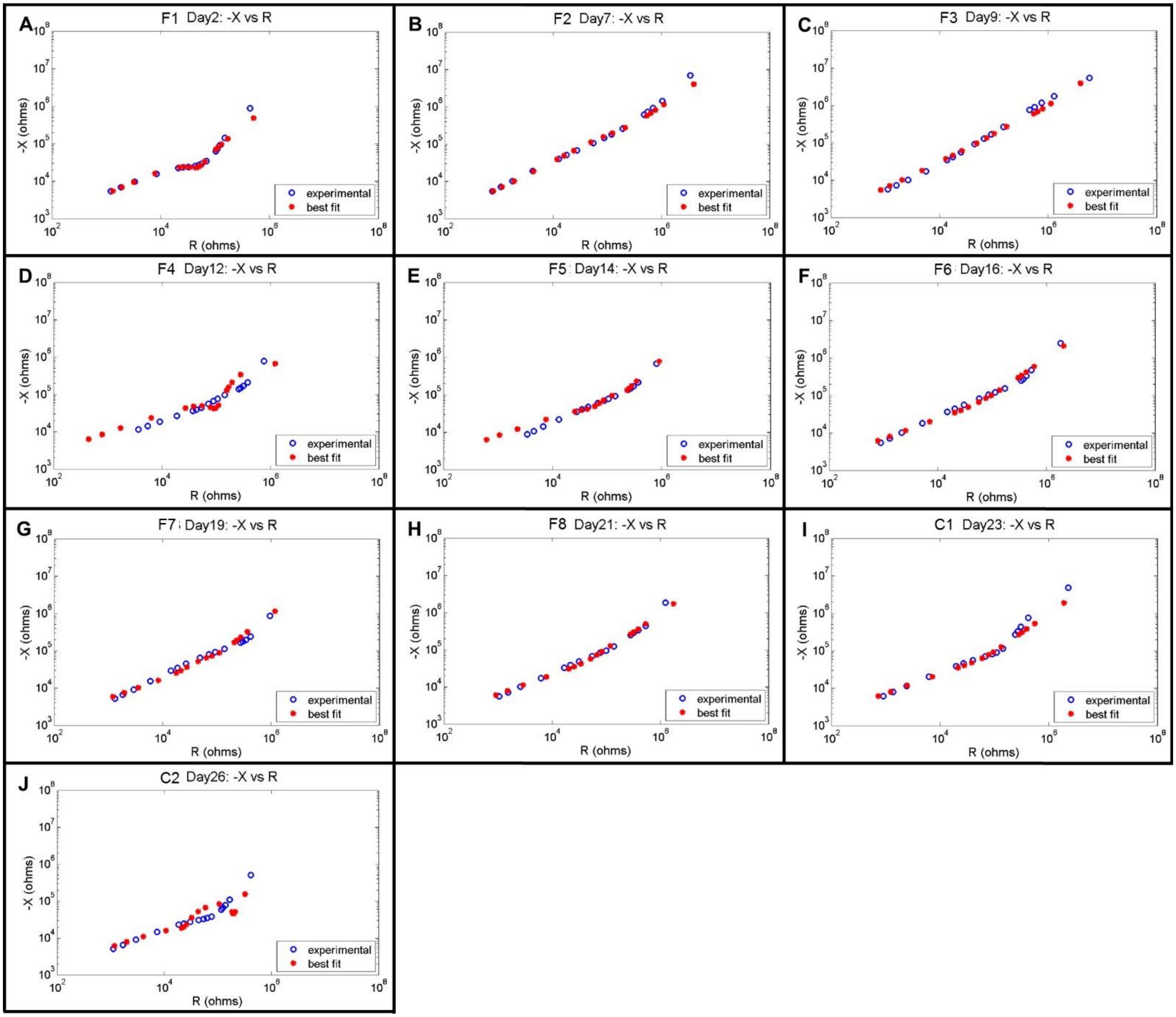

Figure S7 – Goodness of fits for data fit to equivalent circuit model.

Representative set of graphs depicting the experimental values and best fit values derived from the equivalent circuit model of electrical reactance plotted over electrical resistance.

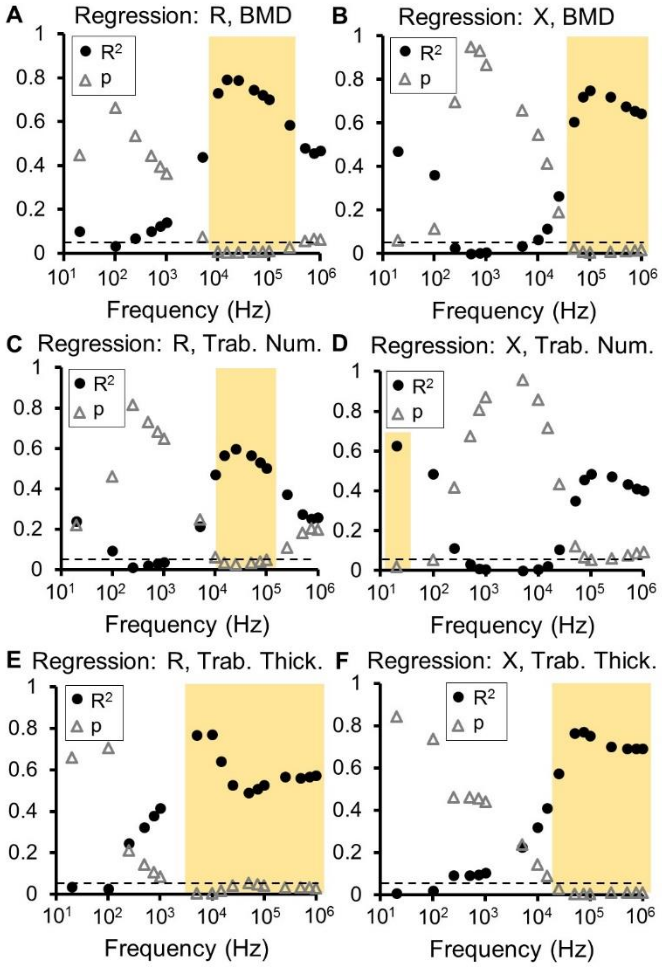

Figure S8 – Clinically-relevant frequencies of operation with significant correlation between impedance data and μCT. Resultant R^2 and p values from regression analyses comparing normalized R and X with bone mineral density (BMD), trabecular number, and trabecular thickness. Significance is set at $p < 0.05$ (below dashed line). Significant relationships are highlighted in yellow.

Table S1 – Stereology of bone plate model samples.

Amounts of cartilage, fibrous, new bone, and new marrow are presented as absolute volumes and as percentages of total callus volume for each sample.

Sample	Absolute Volume (mm^3)					Percentage of Total Callus (%)			
	Total Callus	Cartilage	Fibrous	New Bone	New Marrow	% Cartilage	% Fibrous	% New Bone	% New Marrow
**F1**	6.60	0.95	3.70	1.41	0.54	14.36	56.11	21.31	8.22
**F2**	7.03	1.75	2.81	1.96	0.51	24.88	39.98	27.92	7.22
**F3**	7.07	0.86	4.76	1.23	0.23	12.10	67.26	17.34	3.30
**F4**	4.16	0.10	1.40	1.10	1.57	2.29	33.67	26.33	37.71
**F5**	5.00	0.00	0.71	1.59	2.70	0.00	14.14	31.88	53.98
**F6**	9.98	0.05	2.31	2.38	5.25	0.50	23.11	23.83	52.56
**F7**	7.75	0.53	2.02	2.24	2.96	6.85	26.12	28.88	38.15
**F8**	9.14	0.40	4.05	1.93	2.76	4.43	44.28	21.09	30.20

Table S2 – Modified RUST scores of bone plate model samples.

Five surgeons scored each cortex (excluding the anterior cortex) from 1 to 4: 1 = no callus, 2 = callus present, 3 = bridging callus, 4 = remodeled with no visible fracture line. Scores from the three cortices for each sample were added to generate a total score between 3 and 12. Each surgeon also clinically categorized each sample as union (U), nonunion (NU), or suspected nonunion (SNU).

	Surgeon 1					Surgeon 2				
**Sample**	**Cortex 1**	**Cortex 2**	**Cortex 3**	**Total**	**Category**	**Cortex 1**	**Cortex 2**	**Cortex 3**	**Total**	**Category**
**F1**	1	2	2	5	NU	2	1	2	5	SNU
**F2**	2	2	2	6	NU	2	1	2	5	SNU
**F3**	3	2	2	7	SNU	2	1	2	5	SNU
**F4**	3	2	3	8	U	3	3	3	9	U
**F5**	3	2	3	8	U	3	3	3	9	U
**F6**	3	3	3	9	SNU	3	3	3	9	SNU
**F7**	2	2	2	6	NU	2	2	2	6	NU
**F8**	3	3	3	9	U	3	3	3	9	U

	Surgeon 3					Surgeon 4				
**Sample**	**Cortex 1**	**Cortex 2**	**Cortex 3**	**Total**	**Category**	**Cortex 1**	**Cortex 2**	**Cortex 3**	**Total**	**Category**
**F1**	1	1	1	3	NU	1	2	2	5	SNU
**F2**	1	1	1	3	NU	2	1	2	5	SNU
**F3**	2	1	2	5	NU	2	2	2	6	SNU
**F4**	3	2	3	8	U	3	3	3	9	U
**F5**	3	2	3	8	U	3	3	3	9	U
**F6**	3	3	3	9	U	3	3	3	9	SNU
**F7**	3	3	2	8	NU	2	3	2	7	SNU
**F8**	3	3	3	9	U	3	3	3	9	U

	Surgeon 5				
**Sample**	**Cortex 1**	**Cortex 2**	**Cortex 3**	**Total**	**Category**
**F1**	2	2	2	6	SNU
**F2**	2	1	2	5	SNU
**F3**	2	3	2	7	SNU
**F4**	3	2	3	8	U
**F5**	3	4	3	10	U
**F6**	3	3	3	9	U
**F7**	3	2	3	8	U
**F8**	3	3	3	9	U

Table S3 – Quantified µCT indices for bone plate model samples.
The cortical surface and bone morphology for each sample was assessed from µCT scans in the region between the central surgical screws, and a number of three-dimensional microstructural indices are reported here.

Mouse	Day	Bone Volume (BV)	Total Volume (TV)	BV/TV	Bone Mineral Density (BMD)	Trabecular Number	Trabecular Thickness	Trabecular Separation
**F1**	12	0.2129	2.4666	0.0863	135.8959	3.3514	0.0489	0.3197
**F2**	12	0.1029	2.3747	0.0433	99.1292	2.9957	0.0504	0.3456
**F3**	12	0.1831	2.6416	0.0693	116.7744	2.2935	0.0607	0.4634
**F4**	26	1.7574	4.6154	0.3808	335.406	8.2039	0.069	0.1146
**F5**	26	2.38	5.9458	0.4003	355.0899	9.0805	0.0645	0.1023
**F6**	26	3.8163	16.4001	0.2327	219.9742	7.226	0.045	0.134
**F7**	26	2.6295	9.2405	0.2846	260.3964	7.3311	0.0528	0.1405
**F8**	26	1.8757	7.4606	0.2514	237.9708	7.302	0.0489	0.1401

Table S4 – Resulting R^2 and p values from regression analyses comparing impedance to quantified µCT indices. Regression analyses was performed to compare normalized electrical resistance (R) and reactance (X) to each of the reported µCT indices. The resultant R^2 and p values associated with each relationship are provided with 2 significant digits. If the p value is listed as 0.00, this indicates a value less than 0.01. Significance is set as $p < 0.05$, with significant p values underlined.

Normalized Resistance (R)

	**Freq (Hz)**	**20**	**100**	**250**	**500**	**750**	**1k**	**5k**	**10k**	**15k**	**25k**	**50k**	**75k**	**100k**	**250k**	**500k**	**750k**	**1M**
**BV/TV**	R^2	0.12	0.04	0.06	0.09	0.12	0.13	0.41	0.69	0.76	0.75	0.70	0.68	0.65	0.53	0.43	0.40	0.41
	p	0.41	0.65	0.55	0.46	0.41	0.38	0.09	0.01	0.01	0.01	0.01	0.01	0.02	0.04	0.08	0.09	0.09
**BMD**	R^2	0.10	0.03	0.07	0.10	0.12	0.14	0.44	0.73	0.79	0.79	0.75	0.72	0.70	0.58	0.48	0.46	0.47
	p	0.45	0.66	0.53	0.45	0.39	0.36	0.07	0.01	0.00	0.00	0.01	0.01	0.01	0.03	0.06	0.07	0.06
**Trab. Num.**	R^2	0.24	0.09	0.01	0.02	0.03	0.04	0.21	0.47	0.56	0.60	0.56	0.53	0.50	0.37	0.27	0.25	0.26
	p	0.22	0.46	0.82	0.73	0.68	0.65	0.25	0.06	0.03	0.02	0.03	0.04	0.05	0.11	0.18	0.21	0.20
**Trab. Thick.**	R^2	0.03	0.03	0.25	0.32	0.38	0.41	0.77	0.77	0.64	0.53	0.49	0.51	0.53	0.57	0.56	0.57	0.57
	p	0.66	0.71	0.21	0.14	0.10	0.09	0.00	0.00	0.02	0.04	0.05	0.05	0.04	0.03	0.03	0.03	0.03
**Trab. Sep.**	R^2	0.24	0.09	0.01	0.01	0.02	0.02	0.13	0.29	0.36	0.40	0.39	0.37	0.35	0.27	0.20	0.18	0.18
	p	0.22	0.48	0.84	0.78	0.74	0.71	0.39	0.17	0.12	0.09	0.10	0.11	0.12	0.19	0.27	0.30	0.29

Normalized Reactance (X)

	**Freq (Hz)**	**20**	**100**	**250**	**500**	**750**	**1k**	**5k**	**10k**	**15k**	**25k**	**50k**	**75k**	**100k**	**250k**	**500k**	**750k**	**1M**
**BV/TV**	R^2	0.50	0.39	0.03	0.00	0.00	0.00	0.03	0.06	0.11	0.25	0.58	0.69	0.71	0.67	0.63	0.60	0.59
	p	0.05	0.10	0.67	0.93	0.94	0.88	0.68	0.56	0.43	0.20	0.03	0.01	0.01	0.01	0.02	0.02	0.03
**BMD**	R^2	0.47	0.36	0.03	0.00	0.00	0.01	0.03	0.06	0.11	0.26	0.60	0.72	0.75	0.72	0.68	0.65	0.64
	p	0.06	0.11	0.70	0.95	0.93	0.87	0.66	0.55	0.41	0.19	0.02	0.01	0.01	0.01	0.01	0.02	0.02
**Trab. Num.**	R^2	0.63	0.49	0.11	0.03	0.01	0.00	0.00	0.01	0.02	0.11	0.35	0.46	0.49	0.47	0.43	0.41	0.40
	p	0.02	0.06	0.42	0.68	0.81	0.87	0.96	0.86	0.72	0.43	0.12	0.07	0.05	0.06	0.08	0.09	0.09
**Trab. Thick.**	R^2	0.01	0.02	0.09	0.09	0.10	0.10	0.22	0.32	0.41	0.57	0.76	0.77	0.75	0.70	0.69	0.69	0.69
	p	0.84	0.74	0.46	0.46	0.46	0.44	0.24	0.14	0.09	0.03	0.00	0.00	0.01	0.01	0.01	0.01	0.01
**Trab. Sep.**	R^2	0.64	0.42	0.11	0.03	0.01	0.00	0.00	0.00	0.01	0.06	0.21	0.28	0.30	0.31	0.28	0.27	0.27
	p	0.02	0.08	0.43	0.70	0.83	0.89	0.96	0.90	0.80	0.57	0.25	0.18	0.16	0.16	0.17	0.18	0.19

Table S5 – Experimental study design.

Details for the different models and experiments conducted are presented, describing the number of mice in each group and their measurement/histology time points.

Model	Sensor	Fracture Defect	# of Mice (Total)	# of Mice (Analyzed*)	Measurement Time Points	Histology Time Points
External Fixator	250 µm FR4 sensor pins	2 mm	N=5	N=5	Day 0, 3, 4, 7, 10, 11, 14, 18, 21, 25, 28	Day 3 (N=1), 7 (N=2), 10 (N=1), 25 (N=1)
		0.5 mm	N=6	N=6		Day 4 (N=1), 7 (N=1), 14 (N=2), 28 (N=2)
	56 µm Pt wire sensors	0.5 mm	N=6	N=5	Day 0, 4, 7, 11, 14, 18, 21, 25, 28	Day 28 (N=5)
Bone Plate	Polyimide sensors with Pt electrodes	No defect	N=8	N=2	Day 0, 2, 5, 7, 9, 12, 14, 16, 19, 21, 23, 26	Day 26 (N=2)
		<0.25 mm	N=10	N=8		Day 12 (N=3), 26 (N=5)

*# of mice included in analyses after removing mice with incomplete data due to premature death (unexplained) or broken sensors
